# The social emotional developmental and cognitive neuroscience of socioeconomic gradients: laboratory, population, cross-cultural and community developmental approaches

**DOI:** 10.3389/fnhum.2013.00788

**Published:** 2013-11-18

**Authors:** Kylie Schibli, Amedeo D'Angiulli

**Affiliations:** Neuroscience of Imagery Cognition and Emotion Research Lab, Carleton University, NeuroscienceOttawa, ON, Canada

**Keywords:** neurocognitive processes, socioeconomic status, theoretical neuroscience, stress reactivity, cross-cultural differences, cognitive reserve, physical and mental health

The study of the *socioeconomic neurogradients*—i.e., neural differences corresponding to variations in socioeconomic status (*SES*)—is a neonate area of transdisciplinary and multidisciplinary research within neuroscience; this Research Topic portrays the current status in addressing social inequities and life-span brain development.

To start off, papers focus on development areas that are commonly known to be influenced by SES, including attention, language and literacy, reading, and numeracy. The life-course maturation and development of brain activation associated with these skills is reviewed by Lipina and Posner (2012), with emphasis on the early years. Although neuroplasticity is present throughout the lifespan, critical periods justify the importance of early intervention. Neuroscientists have the responsibility to recognize the many facets of poverty and to adopt an interdisciplinary perspective when using research to inform public policy and interventions. Furthermore, *universal* early intervention is recommended—not just targeting lower-SES children.

D'Angiulli et al. (2012a) examine the emotional/motivational states associated with preadolescent children's performance on an auditory selective attention task, which is then correlated with event-related potentials (ERPs) and electroencephalographic (EEG) techniques to identify brain activity associated with levels of SES. Salivary cortisol and affective self-reports were collected throughout the school day. Although lower- and higher-SES children showed similar behavioral performance, the observed patterns of EEG and ERP differences suggest that children from lower-SES may extend more effortful control to perform similarly to their higher-SES counterparts. Lower-SES preadolescents show a genuine (i.e., unconfounded by “non-cognitive” states) information-processing preference to attend equally relevant and irrelevant environmental cues.

To date, neuroscientific research has seldom addressed the relations between SES, cultural factors, and immigrant/native background on children's moral development—the topics investigated by Caravita et al. (2012). These authors find that children from middle-low SES families perceive transgressions of socio-conventional dilemmas (i.e., social order) more strictly than their middle and middle-high SES counterparts, suggesting that environmental circumstances may lead to more rigid guidelines regarding social conventions of morality, because the repercussions may be viewed as more severe (i.e., social exclusion). Incorporating these findings into neuroscientific research would allow better understanding of which brain processes are implicated in moral decision making.

In a related domain, Gavrilov et al. (2012) examine young children's social development focusing on the role of joint attention. When offered a toy to engage in play with an adult, children (especially girls) from highly traditional and religious families engage in joint attention more frequently than children from families who are less religious and embrace western standards. In families where gender stereotypes are more apparent, girls appear to absorb these roles and are more in tune with socio-cultural expectations. This may be associated with the earlier maturation of the ventral prefrontal cortex among girls. Joint attention might thus be a mechanism that allows children to appropriate cultural values into behavior with the moderating influence of various bioecological levels.

Examining the concept of ecology further, Hackman et al. (2012) explore whether neighborhood disadvantage influences stress reactivity in adolescents as moderated by gender. African-American adolescents participated in a stress-inducing task as researchers monitored stress reactivity and recovery reflected in salivary cortisol levels. Neighborhood disadvantage was inversely correlated with parental education level. Furthermore, concentrated neighborhood disadvantage was correlated with adolescent stress reactivity and recovery. Particularly, boys demonstrated higher cortisol reactivity and steeper recovery. These findings support firstly the importance of discerning gender effects in adolescent neurogradients and, secondly, considering neighborhood disadvantage as important variable for intervention.

The implications of SES research within neuroscience are reviewed by D'Angiulli et al. (2012b), with focus on poverty. Unidimensional definitions of poverty may fail to capture the complexities of poverty as a multifaceted construct. Alternatively, human-rights based approaches (incorporating ecological measures such as access to education, healthcare, and the work market) may be more representative of the child's contextual and experiential position in society, because these provide a more comprehensive understanding of the children's individual status as opposed to their families. Also critically examined are tendencies for researchers to dichotomize children into extreme groups and make assumptions that lead (implicitly or explicitly) to interpretations that children from lower-SES are inherently deficient (*deficit attribution*).

Collins (2012) describes the necessity of incorporating a child's rights perspective when conducting neuroscientific research with children. The central principles of the United Nations Convention on the Rights of the Child (UNCRC) are reviewed as to how they should inform standards for respectful research with children, these include: non-discrimination, best interests, survival and development, and views of the child/participation. Power differentials can lead to unintentional discriminatory practices when conducting research, as children are often viewed as inferior or in need of “repair.” Incorporating a child's rights based-approach forces researchers to critically evaluate how they perceive and treat children, as Collins describes reframing the question of “what is a child?” to “who is a child?”

Calderon-Garciduenas and Torres-Jardon (2012) extend the concept of child's rights by describing the effects of air pollution in Mexico City on brain development. Children severely exposed to air pollution in South Mexico City demonstrated several cognitive deficits, with 56% exhibiting prefrontal white matter MRI hyperintensities. Furthermore, exposed children's autopsies demonstrated that nearly half showed precursors of Alzheimer's disease. However, clearly not all children exposed are at risk of developing neurodegenerative diseases. The role of compensatory mechanisms is described in relation to the many factors impacting children's ability to overcome the effects associated with incremental air pollution exposure. Finally, air pollution exposure is linked with SES, since, lacking the support required for healthy development, low-SES children live in the most hazardous conditions.

Bezo et al. (2012) examine SES and health at the national level, specifically, comparing the relationship between social factors in countries and their impact on physical and mental population health. Through sophisticated statistical modeling, the authors discovered a mediational pathway such that rights and freedoms influenced social capital and SES, leading to improvements in physical and mental health. Namely, in democratic countries where there is more freedom to influence public policy, with governments that are more transparent and less corrupted, citizens feel a sense of trust leading to improvements in SES and health. These findings directly affect life outcomes for children and confirm that higher GDP does not necessarily equate better health, as the link between SES and health is not supported without the mediating effects of rights and freedoms.

Lastly, this Research Topic involves two articles targeting changes over the lifespan. Noble et al. (2012) incorporate a cross-sectional correlational study investigating the association between education and hippocampal and amygdala volume over the lifespan. They obtained data from a standardized database detailing the performance on a memory task along with MRI scans of individuals aged 7–87 years, and discovered that age-related decrease in hippocampal volume is more pronounced amongst individuals with little educational attainment especially after 35 years of age. Such discovery interlocks with the paper that follows.

Using a spatial version of the Stroop task to assess and compare conflict monitoring in older and younger adults, Puccioni and Vallesi (2012) test whether there is an age-related deficit in spatial conflict monitoring independent of priming effects, and whether such deficit in turn is influenced by intelligence, cognitive reserve, and years of education. Although older adults are slower at responding than younger adults on the spatial Stroop task, their accuracy is comparable. This finding supports the theory of a general slowing down effect of aging. Older adults' demonstrate increased difficulty responding to incongruent trials following congruent ones, however, this effect diminishes when higher cognitive reserve exists, suggesting that naturally occurring age-related deficits in attention can be mitigated by rich everyday life experiences.

The variety of articles in this Research Topic truly provide the full spectrum of views on the study of socioeconomic neurogradients today, representing, we believe, comparatively the best examples of research in this field from the full spectrum of methodological and theoretical perspectives.
